# BAND: Behavior-Aligned Neural Dynamics is all you need to capture motor corrections

**DOI:** 10.1101/2025.03.21.644350

**Published:** 2025-03-24

**Authors:** Nina Kudryashova, Cole Hurwitz, Matthew G. Perich, Matthias H. Hennig

**Affiliations:** a School of Informatics, University of Edinburgh; Informatics Forum, 10 Crichton St, Newington, Edinburgh EH8 9AB, United Kingdom; b Zuckerman Institute, Columbia University; 3227 Broadway, New York, NY 10027, United States; c Département de neurosciences, Faculté de médecine, Université de Montréal; Pavillon Roger-Gaudry, 2900 Edouard Montpetit Blvd, Montreal, Quebec H3T 1J4, Canada; d Mila, Quebec Artificial Intelligence Institute; 6666 Rue Saint-Urbain, Montréal, QC H2S 3H1, Canada

## Abstract

Neural activity in motor cortical areas is well-explained by latent neural population dynamics: the motor preparation phase sets the initial condition for the movement while the dynamics that unfold during the motor execution phase orchestrate the sequence of muscle activations. While preparatory activity explains a large fraction of both neural and behavior variability during the execution of a planned movement, it cannot account for corrections and adjustments during movements as this requires sensory feedback not available during planning. Therefore, accounting for unplanned, sensory-guided movement requires knowledge of relevant inputs to the motor cortex from other brain areas. Here, we provide evidence that these inputs cause transient deviations from an autonomous neural population trajectory, and show that these dynamics cannot be found by unsupervised inference methods. We introduce the new Behavior-Aligned Neural Dynamics (BAND) model, which exploits semi-supervised learning to predict both planned and unplanned movements from neural activity in the motor cortex that can be missed by unsupervised inference methods. Our analysis using BAND suggests that 1) transient motor corrections are encoded in small neural variability; 2) motor corrections are encoded in a sparse sub-population of primary motor cortex neurons (M1); and 3) combining latent dynamical modeling with behavior supervision allows for capturing both the movement plan and corrections.

## Summary

Motor cortical neural activity is commonly viewed as a low-dimensional dynamics evolving from the movement preparation state, which explains the most of both neural and behavioral variability. We found that movement corrections to unexpected behavior perturbations do not follow the same pattern, with only a small fraction of neural variability explaining large changes in behavior. We show that capturing both movement planning and corrections requires models that incorporate dynamics and weak behavior supervision. We characterize the bidirectional relationships between motor cortical activity and behavior, identifying neural code for both feedforward and feedback-driven motor control.

## Introduction

Movement planning is a central source of variability for both neural activity and behavior during movement execution [1]. As a result, neural population activity during movement can be approximated with autonomous latent dynamics that unfold from an initial preparatory state [2]. It is hypothesized that during the planning phase, the motor cortex receives control inputs from the thalamus that optimize this initial state in a way that minimizes the prospective motor error [3]. The autonomous dynamics that unfolds from the optimized initial state would orchestrate the sequence of muscle contractions that perform the planned movement in the absence of any unexpected perturbations to behavioral outcomes. Accounting for any unexpected movement perturbations requires continuous feedback-driven control based on sensory input.

**Figure 1:**
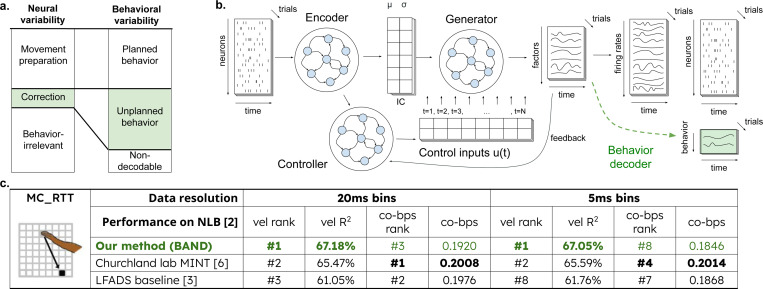
Capturing neural variability that encodes motor corrections. **a)** Hypothetical correspondence between neural and behavioral variability. Small neural variability encodes large behavioral variability of unplanned behaviors; **b)** Our **B**ehavior **A**ligned **N**eural Dynamics model (BAND, black+green), built on top of a sequential autoencoder baseline (LFADS, black), is designed to capture small behaviorally-relevant neural variability. **c)** Validation on Neural Latents Benchmark [6]. The top 3 models with the highest behavior reconstruction quality are shown [7, 5]. Numbers in the table: hand velocity reconstruction (vel $R^{2}$); neural reconstruction on held-out neurons (co-bps [6]); ranks show the position of the model on the NLB leaderboard w.r.t. each metric (excluding ensemble models). BAND has the highest behavioral reconstruction among non-ensemble models but similar neural reconstruction to the LFADS baseline, across all NLB datasets (see all datasets in Fig. S1).

Sensory feedback during movement provides an additional input into the motor cortex during movement execution, which is typically not measured experimentally. These unobserved inputs can be inferred from neural activity as a solution for an optimal control problem [4, 5], provided that they significantly alter neural population trajectory. This assumption, however, might not hold during corrections to perturbed movements, which only transiently deviate from the movement plan. If the deviation in neural dynamics following a perturbation is transient, yet results in motor output, then even a small fraction of neural variability can encode large behavioral responses (Fig. 1a).

In this paper, we develop a method that discovers neural variability that corresponds to movement corrections and demonstrate that movement corrections are indeed encoded in small neural variability. First, we apply our method to hand-reaching datasets in the Neural Latents Benchmark [6], demonstrating that the additional behavioral variability captured by our method corresponds to small neuronal variability. Second, we analyze recordings from the primate motor cortex during a center-out reaching task with an explicit behavioral perturbation, where movement correction can be clearly defined. We provide an in-depth analysis of the neural code for movement corrections and demonstrate that these corrections are encoded in the activity of a sparse sub-population of primary motor cortex (M1) neurons. Finally, we analyse temporal relationships between neural activity and behavior, demonstrating that small neural variability captured by our method comes from sensory feedback that provides motor error signal in perturbation trials.

## Results

We hypothesize that in order to precisely predict trial-to-trial fluctuations in behavior from neural activity in the motor cortex, a model must not only capture the planned component of the behavior, but also corrections and adjustments that occur during movement execution. In the following, we therefore distinguish between planned behavior, which can be decoded from the preparatory neural activity, and unplanned behavior, which requires additional inputs to the motor cortex during movement execution. As we will show, existing models generally fail to predict unplanned behavior, suggesting that it is encoded in much smaller neural variability than planned movement (Fig. 1a).

To predict both planned and unplanned behavior, we introduce the Behavior Aligned Neural Dynamics (BAND) model. BAND employs latent dynamics to model neural population activity, ensuring high-quality neural reconstruction, and, as a secondary objective, aligns latent representation to behavior [8]. BAND is based on the well-established LFADS model which infers latent dynamics and additional control inputs from neural activity (Fig. 1b, black). Importantly, while LFADS is capable of inferring inputs through its controller, this is only the case when these inputs cause a significant change in subsequent neural dynamics, affecting the neural activity reconstruction. A known limitation of LFADS is that its latent space is not well constrained, which can lead to a loss of behaviorally-relevant neural variability. The additional supervision in BAND (Fig. 1b, green + black) ensures that behavior-related neural variability can be captured even if they cause only a small, transient change in neural dynamics.

### BAND captures additional behavioral variability encoded in small neural variability across multiple hand reaching tasks

We first compared BAND to other generative models on three sets of Neural Latent Benchmark recordings from the motor cortex of monkeys performing different variants of hand reach tasks [6]. To compare the accuracy of the models in predicting behavior, we computed the coefficient of determination $\left({\text{R}}^{2}\right)$ to measure the quality of the hand velocity reconstruction. To assess how well the models capture neural variability, we computed the Poisson likelihood and closely-related co-smoothing bits per second (co-bps [6]) as a measures of neural reconstruction quality.

We found that BAND outperformed all other non-ensemble models submitted to the benchmark in terms of the quality of hand velocity reconstruction (Fig. 1c; more datasets in Fig. S1). This consistently high performance of BAND can not be trivially attributed to behavior supervision, since other models (CEBRA [9],MINT [7]) use behavior supervision too. The difference is, however, that BAND combines assumptions about neural dynamics on a lower-dimensional manifold (as in LFADS) with behavior supervision.

Across all models in the benchmark, we found systematic differences in behavior prediction quality between the three hand-reaching tasks. For instance, in a fast-paced random reaching task (MC RTT, shown in Fig. 1c), where one movement directly follows the other without any pre-movement delay periods, reconstruction quality is generally modest. In this case, the LFADS baseline reaches only $R^{2}=62\%$, while it achieves $R^{2}=89\%$ in the center-out reach task (see MC Maze in Fig. S1), which has a clear trial structure. The MINT method from the Churchland lab reaches $R^{2}=65.5\%$ due to its behavior-aware interpolation of latent states [7]. However, MINT still relies on the same AutoLFADS latents, which can limit performance due to the loss of behaviorally-relevant information in AutoLFADS latent space. At the same time, BAND had the largest relative improvement of behavior prediction on this random reach dataset compared to other two datasets (see Supplementary Information Fig. S1), reaching $R^{2}=67\%$. This result suggests that our model is able to capture rapid, transient neural signals that are relevant to behavior, which can be missed by unsupervised latent state inference approaches like LFADS.

Notably, the improved behavior prediction in BAND is not related to improved neural activity reconstruction. The Poisson likelihood achieved by BAND was similar to, but usually slightly below, the LFADS baseline. This was also observed in our previous semi-supervised model [8], and can be attributed to a slight reduction in the model’s ability to capture neural variability not related to behavior. This result suggests that the additional behavioral variability captured by BAND is encoded by small neural variability which has no significant impact on the reconstruction of neural firing.

### Corrections of a perturbed movement are encoded in small neural variability

**Figure 2:**
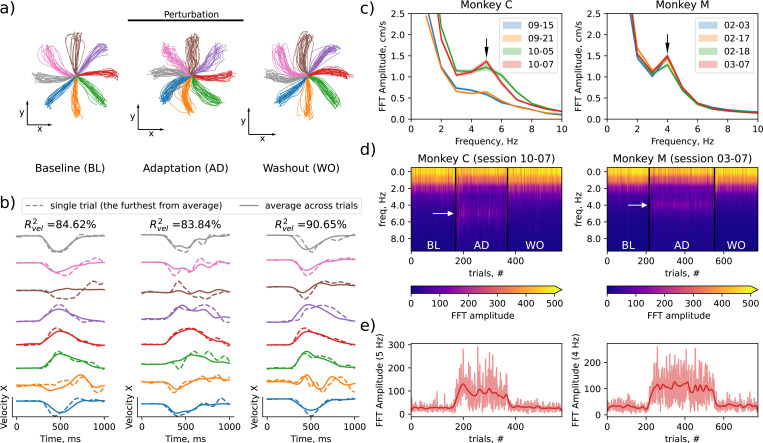
Hand velocity during center-out reaches with a force-field perturbation exhibited 4–5 Hz oscillations. **a)** Hand position during baseline (BL), adaptation (AD) and washout (WO) trials shows the effect of perturbation on hand trajectories **b)** Hand velocity in corresponding epochs; the trials that deviate the furthest from the trial-average velocity for a given condition are shown. **c)** Fourier spectrum of hand velocity in perturbed trials (AD) across recording sessions (color coded) and animals (left and right); arrow shows the peak oscillating frequency. **d)** Fourier spectrum across epochs in the last session for each monkey; white arrow shows the peak oscillating frequency (same as in (c)). **e)** FFT Amplitude of the mode corresponding to the peak oscillating frequency across epochs.

To test the hypothesis that movement corrections are encoded in small neural variability, we analyzed neural recordings from monkeys performing a center-out reaching task with a force field perturbation (Fig. 2a) [10]. In this task, monkeys were required to move a cursor on the screen using a manipulandum, performing reaches to one out of eight specified targets. In perturbed trials, a force was applied on the manipulandum perpendicular to the hand velocity of the monkey. The perturbation caused deviations from a straight trajectory, necessitating online motor correction based on tactile and visual feedback. The experimental session consisted of three epochs: unperturbed Baseline (BL) trials, followed by Adaptation (AD) trials with the perturbation, followed by Washout (WO) trials in which perturbation was removed and the monkey had to re-adapt to normal movements. The trajectories towards the target were straight in BL trials and curved and distorted both in AD and WO trials (Fig. 2a), which indicates online corrections to the planned movement.

### Most behavioral variability in perturbed hand reaches corresponds to the task cue

There are multiple sources of behavioral variability in center-out reaches: variability in target instructions, variability in movement preparation (e.g. errors), and variability in motor execution and online movement corrections. We first tested how much variability can be explained by the target cue alone, in the correctly executed trials (i.e. the cued target is reached). To this end, we calculated the average velocity time course separately for each target direction. This allowed us to account for the behavioural variability between movements towards eight different targets, and provided a baseline for models that only predict this average, but no additional trial-to-trial variability.

Computing the $R^{2}$ measure between this average and the true hand velocity in single trial shows that task cue accounts for a large fraction of behavioural variability. The $R^{2}$ was high across all epochs (Fig. 2b), particularly in the epochs with no perturbation applied ($R^{2}=84.62\%$ and $R^{2}=90.65\%$ in an example session, 86.8±2.8% and 84.3±4.9% across sessions, in baseline and washout epochs respectively). The $R^{2}$ was only slightly lower in the adaptation epoch where force perturbation was applied ($R^{2}=83.84\%$ in an example session, 83.5%±2.2% (mean±std) across sessions). These high $R^{2}$ values suggest that most of variability in hand velocity can be explained by the difference in reach targets in different trials, while only a small fraction of variability (no more than 16.5% ± 2.2% even in adaptation trials) corresponds to other sources of trial-to-trial variability, such as a particular movement correction in response to sensory feedback.

### High-amplitude hand velocity oscillations in response to a force field perturbation

We found that the movement corrections in response to perturbation manifested in high-amplitude hand velocity oscillations, with an amplitude comparable to the overall change in velocity during reaching towards the target (Fig. 2b, velocity in a few adaptation trials is shown in the middle). These oscillations have a period of approximately 200 or 250 ms, or frequency of 4 or 5 Hz, depending on the animal (Fig. 2c). These oscillations appear in adaptation epochs of all recording sessions in both subjects, but vary in magnitude between different sessions (Fig. 2d–e). Data for all sessions are provided in Figs. S2–S3.

### PMd encodes overall hand trajectory while M1 additionally encodes motor corrections

**Figure 3:**
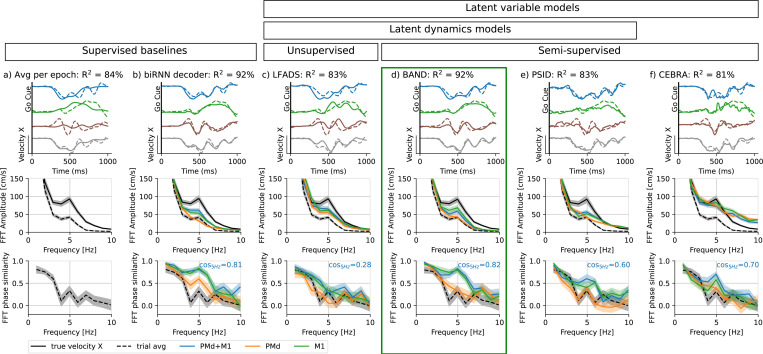
Model comparison shows that both nonlinear latent dynamics and behavior supervision are required for capturing hand velocity. Top row: hand velocity in example AD trials (solid line – model prediction from PMd+M1, dashed – ground truth); middle row: a Fourier spectrum of hand velocity predictions (from both or either brain areas), indicating whether higher amplitude of 5 Hz oscillations is correctly captured; bottom row: cosine similarity between Fourier modes of true velocity vs predicted velocity; cosine similarity at 5 Hz indicates whether the phase of oscillations is captured; **a)** An average velocity towards the reach target (average across all trials with the same reach target within AD epoch); **b)** Velocity predicted by a supervised bi-directional RNN decoder, demonstrating that hand velocity oscillations are decodable from neural activity; **c)** Velocity predicted using ridge regression from LFADS model with a 4-dimensional controller and 100 latent factors; **d)** Velocity predicted by BAND model with 100 factors and all hyperparameters matched to an LFADS model; **e)** Velocity predicted using a kNN decoder from CEBRA embedding; **f)** Velocity predicted by PSID model (a linear Kalman filter with neural and behavioral observations). Decoding here was performed on both PMd and M1 using all epochs, and results evaluated on adaptation epoch; predictions for all reach directions and individual brain areas can be found in Fig. S4)

The presence of strong hand velocity oscillations in some sessions provides the opportunity to investigate whether their neural correlate is found in both M1 and PMd or only in one of these areas. We already established a lower baseline level for decoding performance using a condition-averaged prediction (Fig. 2b and Fig. 3a, $R^{2}=84\%$), which corresponds to the portion of trial-to-trial variability in hand velocities that is explained by the difference in cued reach directions. Condition-average predictions also strongly depend on temporal alignment of trials, which ensures movement phases are synchronized across trials before averaging. Any effects that occur at different times during the trial, such as the hand velocity oscillations, cannot be captured in this trial-averaged model. We performed a Fourier transform on condition-average velocity predictions (Fig. 3a, middle row) to test whether oscillations are phase-locked to the go cue. We observed a large gap between the amplitude of true hand velocity oscillations at 5 Hz and the prediction, suggesting that oscillations were averaged out. In single trials, the phase of the high-frequency Fourier modes (≥ 4 Hz) was also misaligned between true and predicted velocity, as demonstrated by plotting the FFT phase similarity (Fig. 3a, bottom).

We then trained a bi-directional RNN decoder to predict behavior from the neural activity, which takes forward and backward passes through neural data in order to account for closed-loop control of behavior. A backward pass accounts for feedback signal in the motor cortex, allowing the model to decode behavior using information from future, as well as past, neural activity. This supervised approach confirmed that a considerable portion of additional trial-to-trial variability in hand velocity was decodable from neural activity in motor cortices (Fig. 3b, top, $R^{2}=92\%$; more sessions in Table S4). We compared Fourier spectra of predicted hand velocities from different brain areas and observed that the peak at 5 Hz was reconstructed from M1, but not from PMd activity (Fig. 3b, middle row). Even more importantly, the phase of the oscillation was only decodable from M1, but not PMd, as indicated by a difference in cosine similarity ($cos_{5Hz}=0.85$ for M1 yet only $cos_{5Hz}=0.60$ for PMd, Fig. S4 example session 2016–10-07; the effect reproduces in other sessions Fig. S4). This agrees with the expectation that hand velocity oscillations are triggered by sensory feedback, which is processed by M1, but not PMd [11]. While oscillations were decodable from M1, we saw little evidence for single neurons exhibiting the corresponding oscillations (see Supplementary Information text), suggesting that this information is encoded on a population level. The overall direction of movement was decoded from PMd and M1 almost equally well, although the velocity decoding performance was always higher for a combination of two areas (Table S4). These results suggest that PMd and M1 carry complementary information about movement.

Note that not all behavioral variability is decodable for several reasons 1. First, movement execution might imperfectly translate motor commands into movement, adding unpredictable behavioral variability. Second, the observed neural population might not faithfully capture all neural variability in the motor cortex due to undersampling. Therefore, a powerful unconstrained bi-directional RNN oracle model establishes an approximate upper limit of decodable behavioral variability ($R^{2}=91\%$ in this session). Yet, the biRNN decoder does not provide any insight into the neural code for behavior. We therefore compare this top performance to the decoding performance of latent variable models that implement additional constraints on the structure of the neural code.

### Capturing the neural code for movement corrections requires both dynamics and behavior supervision

Behavior decoding with the biRNN shows *what* is encoded in neural population activity, but not *how* this neural code is organized. How much neural variability accounts for movement corrections? Are these corrective hand oscillations initiated by M1, or fed back into M1? To answer these questions, we utilize *latent variable modeling*.

First, we tested whether the information about hand velocity oscillations is preserved in latent dynamics models. These models summarize the activity of the neuronal population in a lower-dimensional set of latent factors. Different models follow different principles and objectives to identify latent variables [12]. Since we hypothesize that movement correction is encoded in a relatively small neural variability (Fig. 1a), we expected that the methods that only rely on neural activity to identify latent variables, without any behavioral signal for supervision, will tend to discard movement corrections as noise. However, since movement corrections cause a substantial change in behavioral output, we expected that adding behavior supervision (Fig. 1b) would ensure that the neural code for movement correction is included in the latent variables.

### Latent dynamics models: unsupervised vs. supervised

We first compared a purely unsupervised model (LFADS) with our semi-supervised model (BAND). To aid further analysis of model components, we restricted the initial condition encoder to receiving only preparatory activity, and the controller having access only to the movement execution phase. This prevented the LFADS model from overusing the controller and enabled learning of the task structure (8 targets arranged on the ring) in the inferred initial conditions (Fig. S9).

We decoded hand velocity from LFADS latent factors using a sequence-to-sequence linear decoder (from [8]; see Methods). For this decoder, we predict the the hand velocity at each time step using the latent factors at all time steps. This decoding approach captures complex temporal relationships between neural activity and behavior. For example, changes in neural activity may influence behavior after a short delay, or alternatively, behavioral outcomes may feed back into neural activity via sensory input. Anticipating a mix of these interactions, we chose a sequence-to-sequence decoder capable of simultaneously accounting for both types of relationships.

Sequence-to-sequence linear decoding from LFADS latent factors was able to capture the overall direction of movement from neural activity, achieving $R^{2}=83\%$ (Fig. 3c). This result is similar to a baseline model that predicts average hand trajectory knowing the true cued reach direction (Fig. 3a, top). Decoding from LFADS latent factors could also predict the presence of the 5 Hz oscillatory mode in hand velocity (Fig. 3c, middle), but not the phase of these oscillations (Fig. 3c, bottom; cosine similarity 0.27). This suggests that unsupervised nonlinear dynamics in LFADS could capture instructed reach direction, but could not explain and correctly time online motor corrections.

We used the same sequence-to-sequence behavior decoder as part of our BAND model, which was, however, trained jointly with the latent dynamics model. Unlike LFADS, BAND accounts for behavior reconstruction in addition to neural reconstruction through semi-supervised training (see Methods). This allowed a BAND model with the same capacity as a corresponding LFADS model to capture motor corrections. BAND achieved high accuracy in behavior reconstruction, $R^{2}=92\%$, matching the biRNN decoder performance in overall hand trajectory reconstruction (Fig. 3d, top) and capturing the phase of the oscillations (Fig. 3d, bottom). Unlike LFADS, BAND can summarize both movement plan and correction in the latent dynamics without a significant loss of neural reconstruction performance (0.264 vs. 0.259 bits / second for the models shown here; standard deviation for cross-validated neural reconstruction is 0.004 bits / second). This difference between semi-supervised and unsupervised models suggests that these online motor corrections are encoded in small neural variability, which is ignored by a purely unsupervised LFADS model which maximizes neural reconstruction quality.

### Linear latent dynamics with a fixed-lag behavior supervision

We next tested whether non-linear dynamics was essential for achieving a high behavior reconstruction for center-out reaching. In theory, the overall hand reaching plan can be represented as a rotation in a latent space [13], which is a linear dynamics mode. The movement corrections to a force field perturbation, as illustrated in Fig. 2, mostly correspond to a 5 Hz oscillation, which is also a linear mode once it is switched on in perturbed trials. Therefore, both important dynamical modes can be represented with linear dynamics, as long as these modes can be switched on and off.

We therefore compared our results with another semi-supervised, yet linear, dynamical model, Preferential subspace identification (PSID [14]). PSID is based on a linear Kalman smoother, which summarizes neural activity into linear combinations of latent factors with linear dynamics. As a Kalman smoother, PSID combines linear dynamic predictions with incoming evidence from the data, which can, in principle, account for nonlinear switches between dynamic modes (e.g. preparation to movement). An additional feature of PSID is that the latent space is split into two: a behaviorally relevant subspace that dissociates and prioritizes behaviorally relevant dynamics, and a behaviorally irrelevant subspace. The behaviorally relevant subspace is assumed to be temporally aligned with the neural activity (possibly, with a fixed lag), which is more restrictive than the linear sequence-to-sequence decoding in BAND.

We first assessed the eigenvalues of the learned dynamics in PSID and found the minimal number of factors that gives rise to latent neural oscillations with a period of 200±100 ms (5 Hz). We found that PSID needs at least 6 behaviorally relevant factors. PSID with only behaviorally irrelevant factors (which is, essentially, an unsupervised Kalman smoother of neural activity) needs >17 factors. If unsupervised PSID is trained on perturbed trials only, then it needs at least 9 factors to predict oscillations. This result suggests that oscillations are encoded in high-order components of a linear state-space model.

However, these 5 Hz oscillations in PSID hand velocity predictions appeared to be out of phase. We tested the behavior reconstruction performance of the PSID model with 100 factors (same as LFADS/BAND). We optimized the number of behaviorally relevant factors to ensure the most accurate decoding of movement in this dataset, resulting in 40 relevant and 60 irrelevant factors. Despite capturing some neural oscillations with the 5 Hz frequency, the overall behavior reconstruction quality was relatively low ($R^{2}=83\%$, Fig. 3e, top), comparable to condition-averaged model ($R^{2}=84\%$, Fig. 3a, top). In general across the adaptation trials, the oscillations were not well-captured, with both the amplitude and the phase of the 5 Hz component poorly predicted (Fig. 3e, middle-bottom rows).

### Supervised but not dynamic latent variable model

Finally, we tested whether behavior supervision alone, without latent dynamics, is sufficient to obtain a latent representation of movement plan and correction. We tested this hypothesis using a recently proposed supervised embedding method CEBRA [9]. CEBRA accounted for some motor corrections, but also added high-frequency features to velocity predictions that are absent from the original signal (Fig. 3f, middle row). CEBRA failed to reproduce a distinct peak in amplitude of 5 Hz oscillation frequency, yet captured the phase of oscillations more accurately than phases in other frequency bands. However, the accuracy of reproducing the phase was still low: the cosine similarity between the true phase and the predicted oscillation phase was 0.66: comparable to PSID (0.61), but substantially lower than that of the biRNN decoder (0.80) or BAND (0.83). This suggests that behavior supervision alone, without explicitly modeling neural dynamics, is insufficient for capturing motor corrections.

### Movement correction is encoded in a small portion of neuronal variability

As discussed above, we restricted the encoders of both LFADS and BAND model in such a way that initial conditions are inferred based on the pre-movement period only and, therefore, have no access to spiking activity during movement. As a result, initial conditions can only account for the movement plan, not the actual executed movement. The controller, on the contrary, could only access spiking activity starting from movement onset, thereby accounting for neural activity during movement, including movement correction.

**Figure 4:**
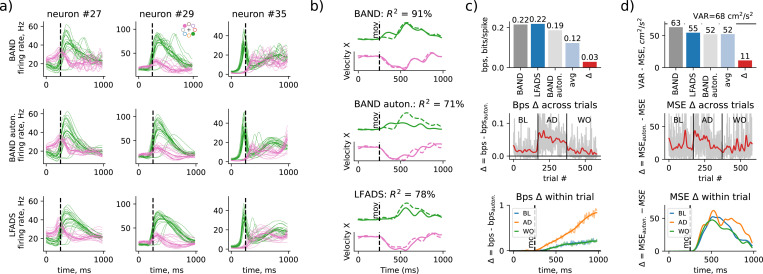
Controller in BAND accounts for movement correction, adding oscillations to latent factors, which make a minor contribution to neural reconstruction, yet a major contribution to behavior reconstruction $\left(R^{2}\right)$. **a)** Control input adds oscillations to predicted firing rates (top row); responses to two example cues for two example neurons are shown; unsupervised baseline model (LFADS, bottom row) does no capture oscillations in firing rates either, despite having a controller. **b)** Behavior reconstruction in a full BAND model (top) is considerably higher $\left(R^{2}=91\%\right)$ than that of an autonomous BAND model (middle, 71%) and unsupervised baseline (bottom, 78%). **c)** A comparison of neural reconstruction quality between the full BAND model (grey), unsupervised baseline model (LFADS, blue), an autonomous BAND model (with ablated controls, light grey) and the neural reconstruction based on the average firing rate (light blue) for every reach direction. The difference between full and autonomous BAND models are shown in red: across the whole dataset (left panel), across different trials and epochs (middle panel) and across time for different epochs (right panel). **d)** Same as (c), but comparison of behavioral reconstruction instead of neural reconstruction. Explained variance (total variance minus mean squared error) is used as a measure of reconstruction quality.

To test whether the controller indeed produces movement corrections, we removed the controller after training, leaving only the autonomous dynamics initiated by the initial conditions (autonomous BAND). As shown in Fig. 4a, the predicted firing rates of M1 neurons differed between the full BAND model and autonomous BAND, with the controller introducing oscillations in the firing rates after movement onset. After ablation of the controller, these oscillations disappeared and the firing rates were qualitatively similar to the firing rates predicted by LFADS. This suggests that behavior supervision in the full BAND model does not affect the autonomous neural dynamics, but instead introduces additional behaviorally-relevant neural variability to the controller inputs.

The quality of behavior prediction decreased significantly when the control input was removed: from $R^{2}=91\%$ to $R^{2}=71\%$ (Fig. 4b). In comparison, linear sequence-to-sequence regression from LFADS factors resulted in $R^{2}=78\%$, which is only slightly better than that of the autonomous BAND model. This result is unsurprising since the autonomous BAND model has a lower capacity in its latent space due to ablation of control inputs and, therefore, is expected to perform worse than LFADS with a controller. The reach direction was still captured after ablation of control inputs, yet the hand velocity oscillations were out of phase, similar to behavior decoded from LFADS factors (Fig. 4b and Fig. S7).

Ablating the controller did not significantly change the neural reconstruction quality (Fig. 4c, left), resulting in only 0.03 bits / second change between full and autonomous BAND models. The autonomous BAND model could still predict much more neural variability compared to a variability captured with condition-average peristimulus-time histograms (avg PSTHs 0.12 bits/spike, Fig. 4c, left). Note that capturing movement correction information in our model did not improve the identification of subsequent latent neural dynamics in comparison to unsupervised LFADS model. This result suggests that autonomous dynamics initiated by preparatory activity could capture a large fraction of neural variability, confirming that preparatory activity is a major source of neural variability [1].

Likewise, preparatory activity was confirmed to be the main source of behavioral variability, accounting for more than 75% of total behavioral variability: the autonomous BAND model accounted for 52 out of 68 *cm*^2^/*s*^2^ (Fig. 4d, left). The condition average behavior accounted for the same amount of behavioral variability as autonomous BAND, suggesting that autonomous dynamics could account for all the task-cue related variability. Yet, there was still a significant fraction of decodable behavioral variability (16%) that was only captured by control inputs in BAND. Therefore, there is a second major source of behavioral variability that was captured by the control inputs (∆ = 11*cm*^2^/*s*^2^ in Fig. 4d, left), that was decoded based on patterns of activity that are relatively small and sparse compared to ongoing neural dynamics and comprise a small fraction of variability in spiking activity during movement (∆ = 0.03 bits / spike in Fig. 4c, left).

We next investigated which trials and which timepoints during the trials were associated with the control inputs. We found that neural reconstruction explained by input-driven dynamics peaked in early adaptation trials and trailed off during the adaptation epoch, suggesting that controller input accounted for responses to force-field perturbation (Fig. 4c, middle). This additional neural variability modelled with the controller follows the same across-trial trend as the power of 5-Hz neural oscillations in hand velocity (see Supl. Fig. S11). The impact of the controller on neural reconstruction was also increasing towards the end of the trial for all epochs (Fig. 4c, right). Behavioral variability followed a similar trend across adaptation trials, yet there were additional unperturbed trials (BL/WO) that had a strong behaviorally-relevant input from the controller. We found that these are the trials in which the execution of the movement was delayed (see Fig. S12). Finally, the behavior prediction was generally most affected by the controller when the hand velocity is the highest (around 500 ms, Fig. 4d, right). Yet, in adaptation trials, the contribution of the controller remains high towards the end of the trial, where behavior is expected to be strongly feedback driven.

Although the contribution of the controller to explaining the observed neural activity increases over time (Fig. 4c), the latent trajectories in BAND were converging back to the autonomous dynamics towards the end of the trial (see Fig. S8). This confirms our hypothesis that control inputs only cause a transient deviation from the autonomous dynamics associated with the planned movement.

### Behavioral variability captured with behavior supervision feeds back into neural activity

To analyse the relationship between neural and behavioral factors we visualized the weights of the BAND behavior decoder. A flexible sequence-to-sequence behavior decoder in BAND (shown in green in Fig 1b) linearly transforms neural factor sequences into the behavioral sequences. As a result, neural factors in one phase of the trial can affect behavior at the other, representing different causal relations between neural activity and behavior: feedforward neural movement control and behavioral outcomes feeding back into neural activity through senses. This causal reasoning requires specific constraints on the inference network: the encoders must not have access to future spiking activity, unlike encoders in the original LFADS model [5]. As a result of such constraint, the inferred latent factors at every time point are based only on the past neural activity, and can not reflect neither the future executed movements, nor the future sensory feedback received by the motor cortex.

**Figure 5:**
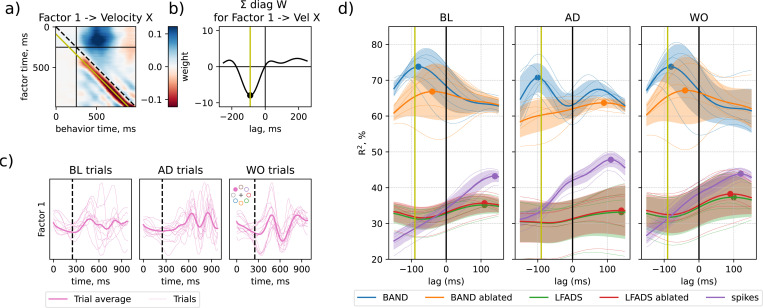
Latent factors in BAND model capture behavioral feedback with a 90 ms lag. a) Behavior decoder weights, transforming a latent factor (here, number 1) into hand velocity (here, component X; see all factor-behavior pairs in Fig. S10). Black vertical/horizontal lines separate preparation and movement phases. Yellow diagonal indicates 90 ms lag between behavior and the latent factor. b) Sum of behavioral weights in (a) across the diagonals in movement phase. c) Factor 1 in baseline, adaptation and washout epochs; 50% of trials are shown, illustrating oscillations in AD trials. d) Fixed-lag prediction of hand velocity (on X-Y plane) from latent state wrt. a variable lag between behavior and latent factors; Negative lag – behavior is leading.

These behavior decoder weights can be represented as a matrix (Fig. 5a). The upper triangle of this matrix corresponds to neural factors causally controlling the behavior, while lower triangle represents behavioral feedback. We visualize the weight matrix after training the model aligned to movement onset time. We note two important features, typical for the weights between all latent neural factors to velocity components (see full model visualization in Fig. S10a). First, there is a concentration of strong positive weights (blue) that connect the initial values of latent factor in the preparatory state (before movement begins at 250 ms) and behavior at about 250 ms after movement onset (500 ms from the start of the trial). These weights correspond to motor planning. Second, there are negative (red) weights on the lower diagonal, which connect hand velocity changes that are slightly lagging behind the neural latent factors. These weights correspond to behavioral feedback.

We then sum the weights along the diagonals of the weight matrices during movement phase (lower right block of the weight matrix, shown in Fig. 5a). Each diagonal correspond to a fixed lag between neural factors and hand velocity. The peak in these weights indicate that changes in hand velocity lag behind neural factors by 90 ms (Fig. 5b). Different latent factors in an 8-factor BAND model contributed to representing feedforward movement planning and behavior feedback to variable degrees: from Factor 7 being mostly feedforward to Factor 4 that mostly reflects behavior feedback (Fig. S10a). Yet, all of the feedback components in these factors are lagging by approximately 90 ms. This result is in agreement with the typical times required for processing sensory input [15] and is slightly slower than 50 ms feedback reported earlier for the reaction to mechanical perturbation in arm movement in monkeys [16].

We then visualized latent factors across baseline, adaptation and washout epochs (Fig. 5c and Fig. S10c). Similarly to our previous analysis of behavior variability modelled with BAND controller (Fig. 4d, right), we observed stronger oscillations in latent factors in the adaptation epoch towards the end of the trial.

We hypothesised that these oscillations are driven by behavioral feedback. To test this hypothesis, we again ablated the controller and analysed how this ablation changes predictions of past and future behaviors. We trained a series of regression models that use the latent state of the BAND model at a fixed time point to predict hand velocities from −150 ms in the past to +150 ms in the future (i.e. computed a multi-variate analog of a cross correlation between neural activity and time-lagged behavioral output). This analysis again demonstrates that latent factors in a full BAND model (blue) are strongly predictive of the behavior at −90 ms in the past, as is expected given the inferred feedback-related weights (Fig. 5b). Ablation of BAND controller flattens the peak almost entirely, suggesting that oscillations in hand velocity are evoked by behavioral feedback that is being processed in the motor cortex.

This analysis also illustrates that both movement planning and feedback were captured in only 8 BAND factors. In comparison, LFADS (with causal constraints on an inference network) achieved significantly lower quality of behavior prediction with the same 8 factors (approximately 35% $R^{2}$ in LFADS vs. more than 60% in BAND with any lag). LFADS also did not show any particular temporal relationship between neural latent factors and hand velocity.

Finally, we applied the same analysis of lags to raw spike trains and found a strong positive peak at around +100 ms. This suggests that there is a strong feedforward control of behavior executed by motor cortical neural activity that causally drives the behavior. Note that there is no positive peak in these plots that could correspond to continual feedforward control in LFADS / BAND models. This is because in these models feedforward motor control is the result of deterministic dynamics, specified entirely by the initial conditions. As a result, a regularized regression model resorts to predicting the whole movement plan from initial conditions, ignoring redundant information in the subsequent dynamics. In contrast, raw spike data is noisy, and regression relies on all timepoints to infer the movement plan and make behavior predictions.

Our results show that movement corrections are driven by sensory feedback, which reaches the motor cortex with an approximately 90 ms delay.

## Discussion

In this work, we found that considerable behavioral variability during hand reaching is encoded in patterns of activity that are relatively small and sparse compared to ongoing neural dynamics and comprise a small fraction of neural variability. In trials with a force-field perturbation, this small neural variability encoded corrections to movement perturbations. We show that the neural activity encoding these movement corrections was feedback-driven, with behavioral changes preceding the changes in neural activity. Finally, we proposed a methodology that allows to capture both feedforward movement planning and feedback-driven correction in a single latent dynamics model.

Our results offer a revised perspective of the dynamical system view of the motor cortical neural activity. While we confirmed the classic result that a major source of both neural and behavioral variability is preparatory activity, we found that the second major source of behavioral variability corresponds to small feedback-driven changes in neural activity during movement. These results are in line with theoretical works that view the primary motor cortex (M1) as a feedback controller [17, 18, 19]. Here we also highlight a new practical implication: this second largest source of behavioral variability requires a specialized data-driven modeling strategy.

We demonstrated that unlike movement planning, feedback-driven movement corrections can not be captured with an unsupervised dimensionality reduction methods. We have shown that behavior reconstruction $\left(R^{2}\right)$ is significantly better in models with behavior supervision, compared to those that discover latent factors in an unsupervised way. The failure of unsupervised dimensionality reduction does not, however, contradict earlier findings that the motor cortical activity is low dimensional [20]. Our BAND model still required very few factors (8 factors in Fig. 5) to qualitatively capture movement planning and corrections. This confirms that the motor cortex operates on a low-dimensional manifold, identified not just by maximizing explained neural variability but also by maximizing explained behavioral variability.

Finally, we provided a strategy for capturing both the movement plan and motor corrections in a latent variable models. We demonstrated that this requires two modeling assumptions: first, it requires dynamics to account for the movement plan and second, it requires behavior supervision to ensure that the neural code for transient movement corrections is captured. We also utilized a powerful sequence-to-sequence decoder for behavior supervision and imposed additional constraints on the model that make the weights of the decoder interpretable. As a result, the BAND parameters allowed us to explore the relationships between neural and behavioral variability based on recorded data, identifying feedforward motor control (motor planning) and lagged feedback-driven motor control.

Capturing behavior feedback in neural activity makes BAND unique among other semi-supervised models. Unlike BAND, the PSID behavior decoder is restricted to predicting behavior that is temporally aligned to latent factors (possibly, with a fixed lag between the two) [14]. A more recent version of this method, dissociative prioritized analysis of dynamics (DPAD [21]), introduced nonlinear dynamics and nonlinear relationships between latent dynamics and behavior, yet retained temporal alignment between time-series as in PSID. While this alignment makes PSID/DPAD easily applicable to timeseries acquired in real time, this limitation prevents these models from capturing bi-directional temporal relationships between neural activity and behavior, which were instrumental for this study (Fig. 5). In contrast, our previous work, Targeted Neural Dynamical Modeling [8], used the same approach to sequence-to-sequence decoding as BAND, yet did not have the controller to capture feedback-driven movement corrections. While the core of the model (RNN generator) can be swapped for more performant architectures [22, 23, 24, 25], the semi-supervised training procedure outlined in this paper should generally allow for capturing movement corrections, since, regardless of the modeling approach, we demonstrated that movement corrections are encoded in small, yet behaviorally-relevant neural variability.

Our analysis was limited to correctly executed reaches in highly trained animals. As a result, we did not consider trial-wise error processing and motor learning that is facilitated by rewards at the end of the trial. Instead, we focused on online, continual error correction. A study by Feulner et al. (2021) [26] considered rapid adaptation to a visuomotor rotation, in which targets on the screen were shifted by 30 degrees. Using modular RNNs, they simulated neural activity changes based on the plasticity of specific modules during adaptation. Their findings suggested that motor adaptation in this context was mediated by upstream areas providing input to motor cortices. A more recent paper from Feulner et al. (2025) [27] linked online corrections and progressive adaptation to changing conditions under a single optimal control framework, yet only for the visuomotor rotation. However, the mechanisms of adaptation to visuomotor and force-field perturbations are likely different [10], since force-field perturbations directly affect the physical forces experienced by the limb, while visuomotor perturbations alter the visual feedback about the movement without changing the actual forces. Another study by Perich et al. (2024) [28] examined the role of feedback in motor learning using a naturalistic task in which monkeys reached for, grasped, and pulled objects mounted on a robotic arm. They found that motor cortex activity was strikingly input-driven surrounding behavioral error correction, and that this input-driven dynamics were isolated in a subspace of the population activity that captured somatosensory feedback. Apart from the analysis, they causally validated their findings with the electrical stimulation of ascending somatosensory tracts. Further analysis with modular biological RNN models and kinematic hand models, as well as causal experimental validation, would be required to localize plasticity in response to force-field perturbation, which is beyond the scope of this paper.

Future work can extend BAND to identify shared latent dynamics in larger datasets with multiple animals, offering an exciting avenue for studying generalizable cross-animal neural representations azabou2023unified, safaie2023preserved, ye2023neural, zhang2025towards, zhang2024exploiting. BAND can also be extended to online real-time applications. While the current analysis was done on structured data, with trials aligned to go cue or movement onset, it can, in principle, be applied to recordings of freely behaving animals and continuous streams of data in real-time (as in [29]). In more naturalistic, unstructured experimental settings we would expect a larger contribution from the feedback-driven control component, and less contribution from feedforward movement planning. In such settings, we would expect an even bigger gap in performance between unsupervised models and supervised models, and we indeed find a bigger gap in the least structured and most fast-paced task analysed here: the random reaching task (MC RTT). In addition, while we analysed pre-recorded data, we applied a causal constraint to the inference network of the controller. This suggests that movement corrections can be captured based only on the past neural activity. Therefore, our modeling approach could also be extended to real-time applications in brain-computer interfaces.

### Neural and behavior reconstruction metrics

The quality of neural reconstruction is measured using bits-per-second (bps) metric [6]:

(1) $$\text{bits}/\text{sec}=\frac{1}{n_{sp}\text{log}2}\underset{n,t}{\sum }\left(𝓛\left({\text{λ}}_{n,t};{\hat{\text{y}}}_{n,t}\right)-𝓛\left({\bar{\text{λ}}}_{n,:};{\hat{\text{y}}}_{n,t}\right)\right)$$

where $n_{sp}$ is the total number of spikes, ${\hat{\text{y}}}_{n,t}$ is the observed spiking activity of neuron n at time t, ${\text{λ}}_{n,t}$ is the rate prediction for neuron n at time t, ${\bar{\text{λ}}}_{n}$,: is the mean firing rate of neuron n, and 𝓛(λ;y) is the Poisson log-likelihood of observed spikes y given rates λ.

Note, that the LFADS/BAND models are trained to explicitly minimize 𝓛(λ;y). The idea behind this metric is to measure how much information about neural firing rates can be explained by the inferred dynamics, discounting trivial prediction of the average firing rate.

The behavioral reconstruction was measured using classic variance explained $R^{2}$:

(2) $$R^{2}(y,f)=1-\frac{\sum_{ntb}{\left(y_{ntb}-f_{ntb}\right)}^{2}}{\sum_{ntb}{\left(y_{ntb}-<y_{ntb}{>}_{nt}\right)}^{2}}$$

Here y is a behavioral variable (hand velocity for hand reaching tasks) and f is a model prediction; $\text{<}y{>}_{nt}$ indicates an average of y over trial n and time t dimensions; b and d correspond to the behavioral component and target direction indices, respectively.

Note, that the $R^{2}$ is often computed for each behavioral component separately and then the average $<.{>}_{b}$ is taken (perhaps, due to the fact that such averaging over features is standard for sklearn library). This ignores the fact that components of the velocity belong to the same 2D Euclidean space. The difference between the resulting scores between our isotropic 2D $R^{2}$ and $<.{>}_{b}$ variant was negligible for the data and models presented in this paper.

In the center-out reach task, $R^{2}$ is dominated by task instruction and is insensitive to the uninstructed variability. Indeed, even the simplest baseline model that uses trial average behavioral trajectory for any given direction $\left(f_{n_{d}tb}=<y_{n_{d}tb}{>}_{n_{d}}\right)$ scores $R^{2}=84\%$ even on the adaptation trials, and $R^{2}=91\%$ on unperturbed trials (see Fig. 2b).

### Datasets with a force field perturbation

We have used 8 sessions with force field perturbation recorded from 2 Rhesus macaque monkeys (Monkey C and Monkey M) from Perich et. al. 2018 [10]. Each session consists of three epochs: baseline (BL), adaptation (AD), and washout (WO).

All sessions were used in our analysis. A few trials were excluded where velocity readings were corrupted and included unrealistically high (>1 m/s) numbers: 3 trials in total, 1 per session in sessions 2016–09-15, 2016–09-21, 2014–02-03.

We used 80% / 20% split between train and test sets to train and evaluate all decoders and latent variable models. We used a single random 80 % / 20% split to benchmark models (Fig. 3), and a 5-fold cross-validation to analyze BAND-model predictions across all trials (Fig. 4–5).

### Fourier analysis of hand velocity and neural oscillations

We applied Fast Fourier Transform (FFT) to the hand velocity to quantify 4–5 Hz oscillations observed empirically in adaptation trials in Fig. 2b. We applied FFT to every component of the velocity in every trial, obtaining powers (squared amplitude) of different Fourier harmonics. We then calculated the mean power across trials for each Fourier mode (shown in Fig. 2)c).

We then applied a similar procedure to the firing rates of the single neurons in PMd/M1. The firing rates were estimated using a Gaussian filter with σ=30ms. FFT was applied to every trial separately, and then the trial average amplitude of each FFT mode was calculated. The neurons were considered oscillatory (see Table S1) if the power of 4 Hz or 5 Hz FFT modes (the ones closest to 4 Hz and 5 Hz) in adaptation but not baseline trials was higher than mean + 4SD of the power distribution obtained from shuffled data. For each trial, spike counts were independently shuffled 500 times. The firing rate and FFT were then recomputed for each shuffled dataset. This criterion specifically identified neurons exhibiting 4–5 Hz oscillations *induced* by the perturbation, excluding neurons oscillating at these frequencies regardless of trial type. In other words, the amplitudes of the 4–5 Hz modes were required to fall within the chance distribution during baseline trials but significantly outside this distribution during adaptation trials.

### Models of motor cortical activity and behavior decoders

#### Average hand trajectory per target

The average hand trajectory per target was calculated on the training set, containing 80% of trials. The variance explained by this average hand trajectory was evaluated with the $R^{2}$ (see eq.2) on the remaining 20% trials (Table S2). These values quantify how much variability in hand velocity is explained by the difference in the reach target.

#### Bidirectional RNN decoder

We used a bidirectional RNN decoder in order to test whether hand velocity oscillations in response to perturbations are decodable from neural populations (M1, PMd, or both areas together). The architecture and hyperparameter values can be found in Table S3.

#### CEBRA and a kNN decoder

We used a CEBRA [9] offset10-model to obtain the 100-dimensional embeddings of neural activity. We used batch size of 512, learning rate 10^−4^ and temperature 1, and trained the model for 10000 iterations with the cosine similarity as the distance metric. We used velocity as a continuous label for CEBRA embeddings.

We then used kNN regression to predict hand velocity, and the number of neighbors k was searched over the range [1, 2500] (same as in the original CEBRA[9]). We fine-tuned the number of neighbors for each dataset based on cosine similarity.

#### PSID decoder

We used PSID model [14] with 100 factors. We optimized the dimensionality of behaviorally-relevant subspace to achieve the highest behavior decoding performance $\left(R^{2}\right)$, which resulted in 40 factors, leaving 60 behaviorally-irrelevant factors.

To make behavior predictions, we used the fixed-time linear behavior decoder from the PSID model. This decoder takes the latent state at a given moment in time and predicts behavioral state at the same time.

#### LFADS as a baseline latent variable model

We used a PyTorch implementation of LFADS model [5] with 100 latent factors, 4 control inputs, 200 generator factors, and 64 encoder dimensions for both initial conditions and controller encoders. The hyperparameters were optimized according to an AutoLFADS [30] as described below.

#### Sequence-to-sequence Ridge regression decoder

After LFADS model was trained, we trained a sequence-to-sequence Ridge regression decoder to decode hand velocity. The latent factors (dimensions: [trials, time, factors]) were flattened as [trials, time x factors]. As a result, every trial constitutes a sample, and the whole latent trajectory provides features for the decoder.

#### BAND: Behavior aligned neural dynamics

We propose a semi-supervised latent dynamics model called Behavior Aligned Neural Dynamics (BAND). The main idea of the model is to use a combination of neural decoder and behavior decoder to ensure that the latent space retains the most behaviorally-relevant neural variability. This idea is related to previously proposed PSID [14] and TNDM [8] methods, in which latent space was split into behaviorally-relevant and behaviorally-irrelevant parts. Yet, BAND does not make such separation, since it aims to retain rather than isolate behaviorally-relevant variability.

Using a well-established latent dynamics model (LFADS, Fig. 1b, black) as a baseline, we constructed a model that not only explains neural variability but also aligns the latent space with the behavioral output (Fig. 1b, green + black) using an additional sequence-to-sequence behavior decoder (Fig. 1b, green). The whole BAND model including the decoder was trained end-to-end.

The negative loss of the model is the following:

(3) $$𝓛={𝓛}_{x}+\theta {𝓛}_{b}-\alpha {𝓛}_{L2}-\beta_{KL_{g0}}{𝓛}_{KL_{g0}}-\beta_{KL_{u}}{𝓛}_{KL_{u}}$$

(4) $${𝓛}_{x}={\langle \sum_{t}\text{log}\left(\text{Poisson}\left({\text{s}}_{t}|{\text{f}}_{t}\right)\right)\rangle }_{{\text{g}}_{0},{\text{u}}_{1:T}}$$

(5) $${𝓛}_{b}=-\text{MSE}(\text{b},\hat{\text{b}})$$

where s are observed spikes, f is a predicted firing rate; ${\text{g}}_{0}$ and ${\text{u}}_{1:T}$ are latent variables: dynamics initial condition and inputs; b and $\hat{\text{b}}$ are the observed and predicted behaviors, respectively; θ is the weight of the behavior supervision term.

The exact value of θ sets the balance between prioritizing neural vs. behavioral reconstruction. If θ is set to a large value, it can cause the model to split the latent representation into mostly independent behavioural and neural subspaces. If θ is set to a small value, then the results are no different from LFADS, which is equivalent to θ=0. The intermediate values, fine tuned with hyperparameter optimization (as described below), result in high neural reconstruction and high behavioural reconstruction quality, with latent factors representing both modalities.

### Constraining LFADS and BAND for interpretability

To achieve interpretable latent representation for a centre-out reaching dataset, we fixed the coefficients β in (3) to 1. With these parameters fixed, the loss function yields a lower bound estimator (ELBO) [31], which means that the posterior distribution of the inferred latent approximates the true posterior. We fixed these coefficients because the goal of our work was the interpretation of movement corrections. Therefore, we wanted to infer initial conditions that encode the movement target and control inputs that correspond to movement corrections during the trial. Without constraining β=1 the Auto-LFADS and Auto-BAND models converged to solutions with $\beta_{KL_{g_{0}}}∼10^{-5}$ and $\beta_{KL_{u}}∼10^{-7}$, and low variability in control inputs. This resulted in overuse of the controller, leading to input-driven dynamics which could not predict planned behavior (i.e. a reach towards the target) without the control inputs. Thus, to avoid this solution, we fixed $\beta_{KL_{g_{0}}}=1$ and $\beta_{KL_{u}}=1$. In addition, we ensured that the encoder for initial conditions can only access the first 25 bins of the trial, while the encoder for control input had access to the rest of the spike sequence (bin 26 and further). This procedure ensures that initial conditions can not contain information on the sensory feedback during movement. Finally, we forced the controller encoder to be *causal*, meaning that at every time step it has no access to future spiking activity.

### Hyperparameter optimization

We used population-based training (PBT, from AutoLFADS [30]) for optimizing hyperparameters. For NLB challenge, the procedure remained close to Auto-LFADS: the behavior weight was fine-tuned in addition to the standard tuned parameters.

We used a normalized neural log likelihood (4) and behavioral $R^{2}$ for all the further analysis in the paper:

(6) $${𝓛}_{PBT}=\frac{{𝓛}_{x}}{N\cdot T}-\left(1-{\text{R}}^{2}(b,\hat{b})\right)$$

where N is the number of neurons and T is the number of time bins in a trial.

#### Adapting a model for NLB challenge

We modified BAND decoder and objective in order to match the target metrics in NLB challenge. We used a standard linear decoder, instead of a sequence-to-sequence behavior decoder, to reproduce NLB decoding procedure. We also used the first two terms in BAND loss in (3) as a PBT objective for NLB challenge, with θ fixed to 10^−4^:

(7) $${𝓛}_{PBT-NLB}={𝓛}_{x}-10^{-4}\cdot \text{MSE}(\text{b},\hat{\text{b}})$$

Both PBT losses in (6) and (7) are equivalent up to constant coefficients before neural and behavioral terms.

The authors would like to thank Juan Gallego, Lee Miller and Robyn Greene for valuable discussions. This research was carried out with the support of the Royal Society University Research Fellowship awarded to N.K. (URF\R1\241060), BBSRC grant (BB/X01861X/1) awarded to M.H.H., grant “chercheurs-boursiers en intelligence artificielle” from the Fonds de recherche du Quebec Santé awarded to M.P. The work of C.H. was supported in part by NIH 5U19NS107613, Simons Foundation (543023), and NSF (DBI-2229929).

## Supplementary Material

Supplement 1
