# Supplementary material for: BAND: Behavior-Aligned Neural Dynamics is all you need to capture motor corrections
Source: bioRxiv. 2025 Mar 24:2025.03.21.644350. Preprint. [Version 1] doi: 10.1101/2025.03.21.644350 (PMC11974739; doi:10.1101/2025.03.21.644350)
Supplement: Supplement 1 [file media-1.pdf]

Weak behavior supervision for latent dynamics is all you need to capture motor corrections

Kudryashova, Nina; Hurwitz, Cole; Perich, Matthew, and Hennig, Matthias H.

March 20, 2025

Hand velocity oscillations are sparsely encoded in neural oscillations in M1 but not PMd

We then used the Fourier transform and searched for a 4-5 Hz oscillations in neural firing rates of PMd and M1 neurons. We found a very small fraction of neurons with significant 4-5 Hz component in M1 (4±1%), and very few such neurons in PMd (0.2±0.5%, see Table 1). Most of the sessions did not contain any recorded oscillating PMd neurons. This suggests that M1 neurons have neural dynamic modes at a relevant timescale, and therefore the phase of oscillations in these individual M1 neurons might encode the phase of hand velocity oscillation.

Since the fraction of such neurons is small, this provides little evidence for single neurons capturing the observed oscillations. As we show in the paper based on the decoding analysis, the information about the phase and intensity of hand velocity oscillations is present in the M1 population code.

MC_Maze	Data resolution	20ms bins				5ms bins			
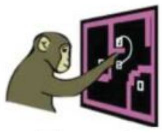	**Performance on NLB [2]**	vel rank	vel R^2	co-bps rank	co-bps	vel rank	vel R^2	co-bps rank	co-bps
	**Our method (BAND)**	**#1**	**92.52%**	#3	0.3537	**#1**	**93.62%**	#14	0.3215
	CEBRA-behavior [4]	#2	91.06%	#9	-1.3571	–		–	
	Churchland lab MINT [6]	#3	90.05%	#4	0.3295	#2	91.21%	#12	0.3304
	LFADS baseline [3]	#4	89.06%	**#2**	**0.3554**	#5	90.99%	**#10**	**0.3364**
**MC_RTT**	**Data resolution**	**20ms bins**				**5ms bins**			
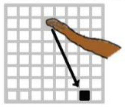	**Performance on NLB [2]**	vel rank	vel R^2	co-bps rank	co-bps	vel rank	vel R^2	co-bps rank	co-bps
	**Our method (BAND)**	**#1**	**67.18%**	#3	0.1920	**#1**	**67.05%**	#8	0.1846
	Churchland lab MINT [6]	#2	65.47%	**#1**	**0.2008**	#2	65.59%	**#4**	**0.2014**
	LFADS baseline [3]	#3	61.05%	#2	0.1976	#8	61.76%	#7	0.1868
**Area2_Bump**	**Data resolution**	**20ms bins**				**5ms bins**			
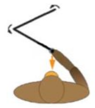	**Performance on NLB [2]**	vel rank	vel R^2	co-bps rank	co-bps	vel rank	vel R^2	co-bps rank	co-bps
	**Our method (BAND)**	**#1**	**88.92%**	#4	0.2477	**#1**	**89.50%**	#8	0.2686
	Churchland lab MINT [6]	#2	88.03%	**#1**	**0.2718**	#2	88.77%	**#7**	**0.2735**
	LFADS baseline [3]	#4	85.65%	#3	0.2542	#10	84.92%	#10	0.2569

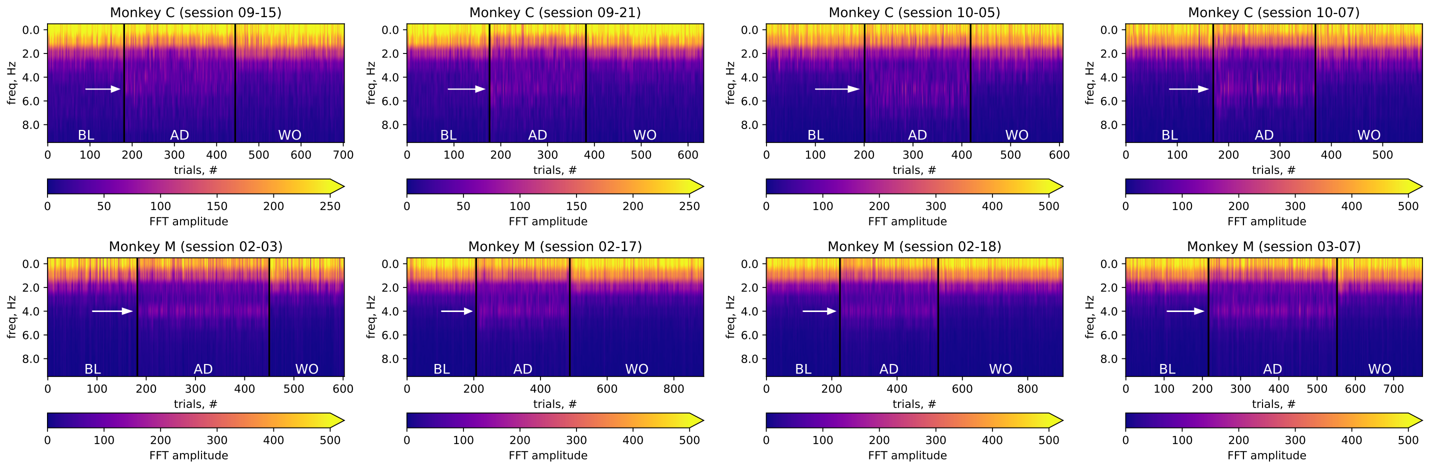

Figure S 2: Fourier spectra of hand velocity across trials and epochs, for both monkeys and all recording sessions. The white arrow points at the oscillatory frequency (5 Hz for Monkey C, 4 Hz for Monkey M), which appears in adaptation trials.

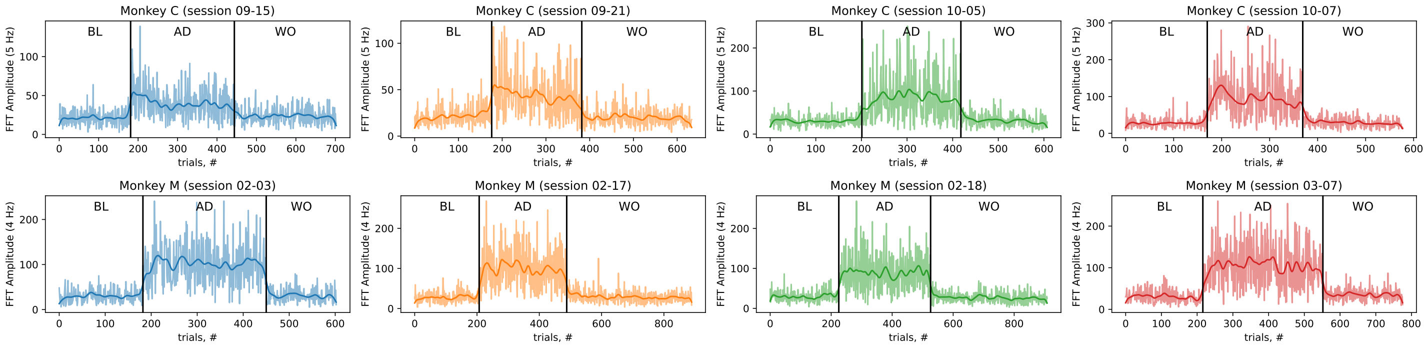

Figure S 3: Fourier amplitude of hand velocity oscillation across trials and epochs, for both monkeys and all recording sessions.

Table S 1: Oscillating neurons in M1 vs PMd

Monkey	Date	M1 neuron ids	PMd neuron ids	M1 count	PMd count
Chewie	2016-09-15	[15, 30, 31, 45]	[157]	4	1
Chewie	2016-09-21	[3, 27, 51]	[]	3	1
Chewie	2016-10-05	[30, 44]	[]	2	0
Chewie	2016-10-07	[1, 27, 23, 35]	[144, 157]	4	2
Chewie	total			13	4
Mihili	2014-02-03	[13]	[51]	1	1
Mihili	2014-02-17	[17]	[]	1	0
Mihili	2014-02-18	[5, 15]	[]	2	0
Mihili	2014-03-07	[2]	[]	1	0
Mihili	total			5	1

Table S 2: Variance explained by an average hand velocity towards a given target (R^2, %)

Monkey	Date	R^2_{all}	R^2_{BL}	R^2_{AD}	R^2_{WO}
Chewie	2016-09-15	85.3	89.9	82.5	83.4
Chewie	2016-09-21	85.9	87.6	82.6	86.4
Chewie	2016-10-05	89.0	90.8	86.1	90.0
Chewie	2016-10-07	86.8	84.6	83.8	90.7
Mihili	2014-02-03	79.9	82.8	81.1	74.4
Mihili	2014-02-17	81.8	83.9	81.2	80.6
Mihili	2014-02-18	86.9	88.8	88.0	84.9
Mihili	2014-03-07	84.1	85.9	82.6	83.9

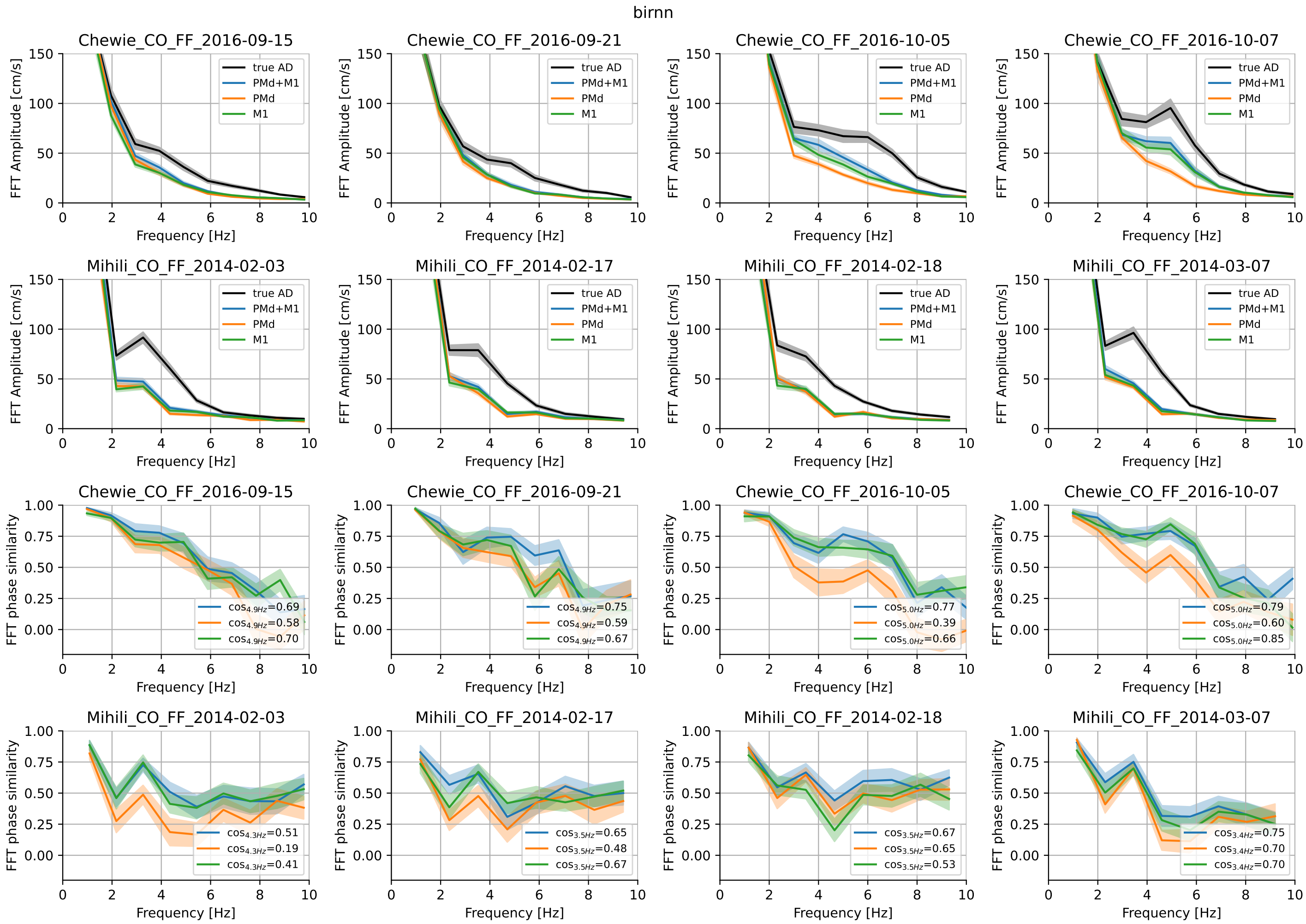

Figure S 4: Fourier spectrum for velocity predictions based on biRNN decoder. In the sessions where hand velocity oscillations are decodable, they are decodable from M1 and not PMd. Note, that the number of M1 neurons recorded in Monkey M is considerably lower then in Monkey C. Brain areas used in the decoding are color-coded (blue: PMd+M1, orange: PMd, green: M1).

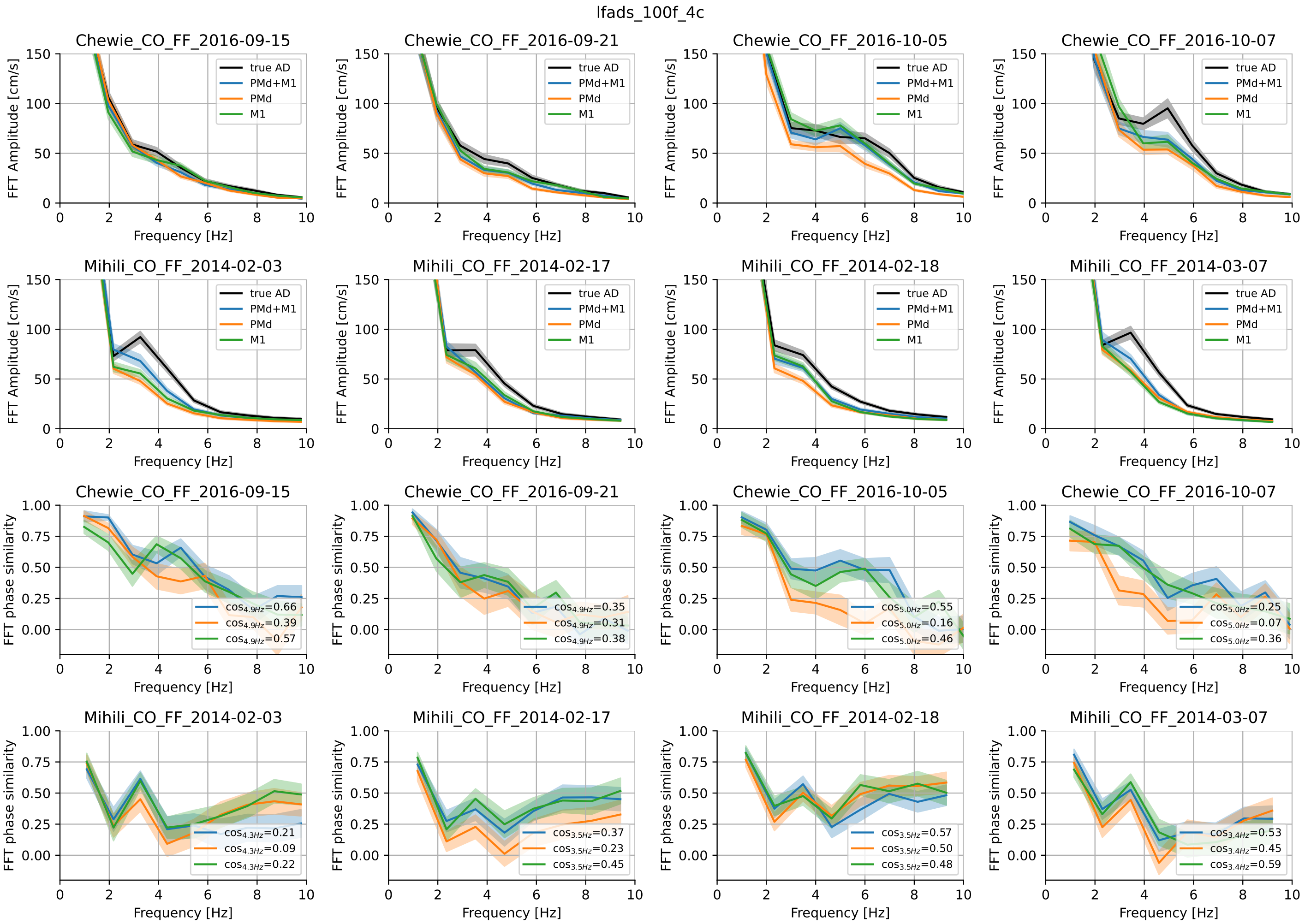

Figure S 5: Fourier spectrum for velocity predictions based on LFADS models with 100 factors.

**Layer**	**Size / Operation**
Input Dropout	Dropout with rate 0.3
Bidirectional LSTM	Input size: number of neurons<br>Hidden size: 128<br>Number of layers: 1<br>Output size: 2×128
ReLU Activation<br>Batch Normalization	-<br>-
Fully Connected (FC) Layer 1	Input size: 256<br>Output size: 128
ReLU Activation<br>Batch Normalization	-<br>-
Dropout	Dropout with rate 0.2
Fully Connected (FC) Layer 2	Input size: 128<br>Output size: number of behavior components (2)

Table S 3: Summary of the biRNN Decoder Network Architecture

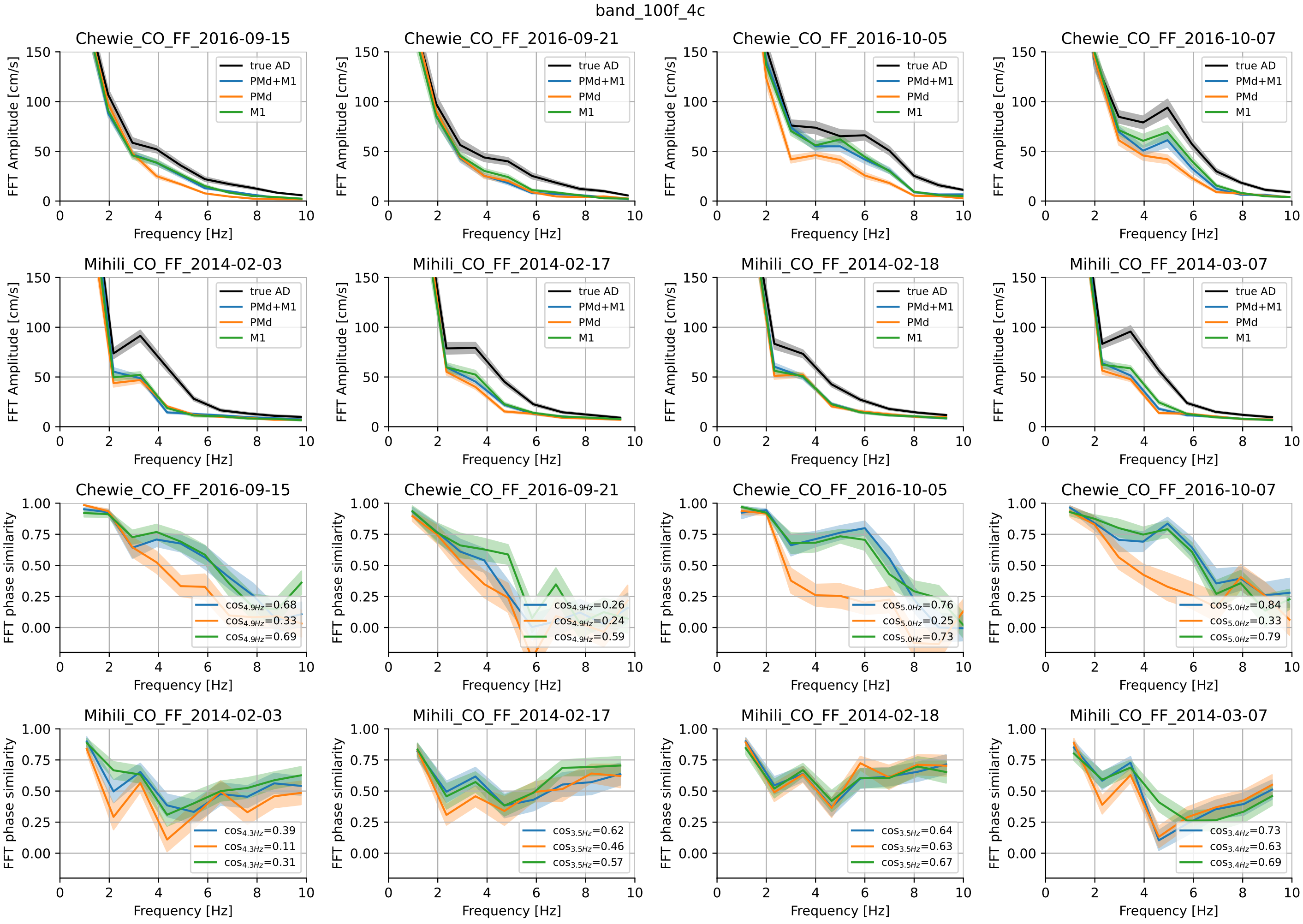

Figure S 6: Fourier spectrum for velocity predictions based on BAND models with 100 factors.

Monkey	Date	both_R^2_{all}	both_R^2_{BL}	both_R^2_{AD}	both_R^2_{WO}	PMd_R^2_{all}	PMd_R^2_{BL}	PMd_R^2_{AD}	PMd_R^2_{WO}	M1_R^2_{all}	M1_R^2_{BL}	M1_R^2_{AD}	M1_R^2_{WO}
Chewie	2016-09-15	93.2	94.6	90.8	93.9	91.1	92.8	88.3	91.6	91.1	93.1	87.8	91.8
Chewie	2016-09-21	92.6	93.9	88.7	93.9	90.2	91.7	85.0	91.9	90.2	91.4	86.3	91.5
Chewie	2016-10-05	94.9	96.2	92.9	95.5	91.2	93.0	87.7	92.7	93.6	95.3	91.0	94.6
Chewie	2016-10-07	94.2	94.6	92.0	95.3	90.0	92.0	84.6	92.3	93.2	93.0	90.8	94.9
Mihili	2014-02-03	85.6	85.9	85.1	84.8	73.6	73.9	75.2	70.2	82.1	82.1	81.0	81.7
Mihili	2014-02-17	91.8	93.5	88.7	92.2	84.4	87.8	81.4	83.8	85.6	86.4	83.3	86.0
Mihili	2014-02-18	91.8	92.2	89.4	92.8	87.5	90.3	84.7	87.3	86.8	88.3	82.1	88.4
Mihili	2014-03-07	87.8	89.0	85.3	88.7	82.5	84.2	80.2	82.3	83.7	85.6	81.1	83.8

Table S 4: Variance explained by a biRNN decoder given neural recordings from M1, PMd, or both areas together (R^2, %). Red indicates cases in which biRNN decoding captured less variance than a model that only predicts reach direction (i.e. R^2 is below that in Table 2).

Monkey	Date	both_R^2_{all}	both_R^2_{BL}	both_R^2_{AD}	both_R^2_{WO}	PMd_R^2_{all}	PMd_R^2_{BL}	PMd_R^2_{AD}	PMd_R^2_{WO}	M1_R^2_{all}	M1_R^2_{BL}	M1_R^2_{AD}	M1_R^2_{WO}
Chewie	2016-09-15	84.3	84.4	80.9	86.3	79.0	80.6	75.0	80.3	66.7	71.1	49.8	75.0
Chewie	2016-09-21	79.3	80.5	73.4	81.4	75.1	77.3	70.0	76.0	67.6	69.1	59.5	70.8
Chewie	2016-10-05	84.1	85.2	81.1	85.8	75.1	76.0	71.7	77.6	76.7	80.2	72.3	77.2
Chewie	2016-10-07	82.8	80.7	80.6	85.9	72.6	74.3	67.6	74.6	71.7	66.8	71.0	75.4
Mihili	2014-02-03	58.7	57.9	58.2	56.6	30.0	33.5	19.4	31.5	43.8	49.7	38.4	36.9
Mihili	2014-02-17	71.8	76.3	67.4	70.9	52.4	59.3	46.3	50.6	54.5	60.5	45.6	55.1
Mihili	2014-02-18	73.4	72.6	72.6	73.5	58.6	58.2	59.2	57.2	55.8	56.8	52.5	56.3
Mihili	2014-03-07	68.3	67.9	69.0	66.0	42.3	37.9	37.2	48.7	49.5	50.2	50.8	44.7

Table S 5: Variance explained by a CEBRA + kNN decoder given neural recordings from M1, PMd, or both areas together (R^2, %). One green value indicates cases in which CEBRA explained more variance than the average hand trajectory (i.e. Table. 2).

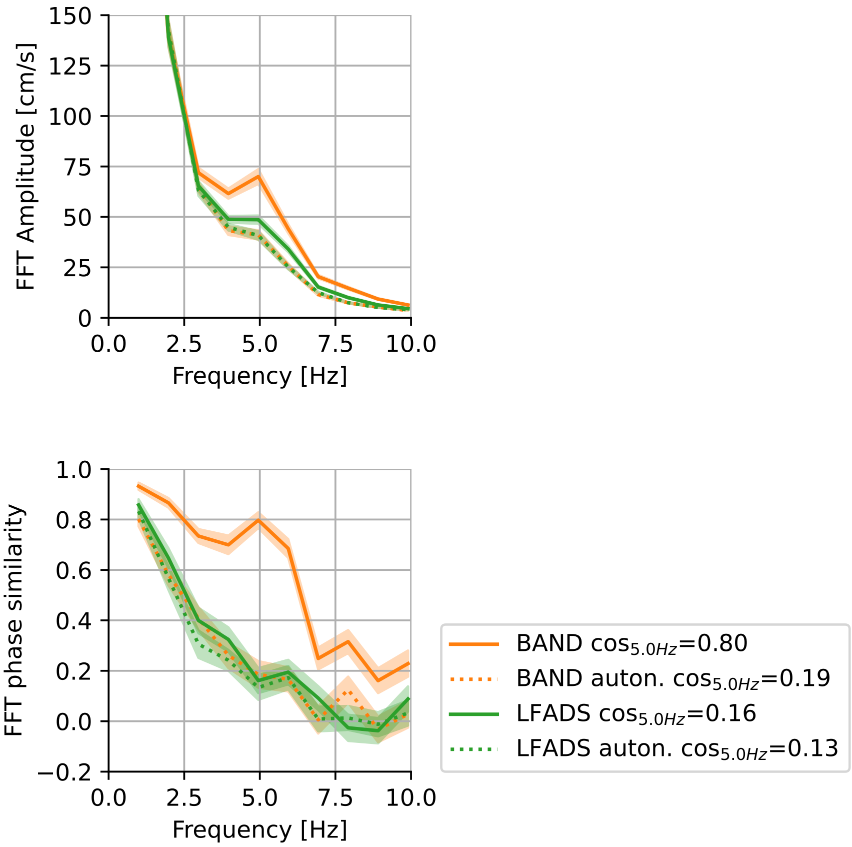

Figure S 7: Fourier spectrum for velocity predictions based on LFADS/BAND models with 100 factors and 4 control inputs (solid) compared to the same models with ablated controller inputs (dashed; autonomous models). Similarly to Fig. 3 in the main text, top plot: a Fourier spectrum of hand velocity predictions (from different models), indicating whether higher amplitude of 5 Hz oscillations is correctly captured; bottom plot: cosine similarity between Fourier modes of true velocity vs predicted velocity; cosine similarity at 5 Hz (in the legend) indicates whether the correct phase of oscillations is captured; *Note: data was aligned to go cue here, as in Fig. 3.; the results are qualitatively the same for movement onset alignment, yet all peaks are less pronounced (not shown).* Considerably higher cosine similarity for our BAND model with a controller indicates that the role of the inferred control inputs in BAND is to correctly capture the phase of hand oscillations.

Monkey	Date	both_R^2_{all}	both_R^2_{BL}	both_R^2_{AD}	both_R^2_{WO}	PMd_R^2_{all}	PMd_R^2_{BL}	PMd_R^2_{AD}	PMd_R^2_{WO}	M1_R^2_{all}	M1_R^2_{BL}	M1_R^2_{AD}	M1_R^2_{WO}
Chewie	2016-09-15	82.9	85.2	78.1	84.4	77.8	80.7	74.1	78.2	78.0	81.7	66.4	83.1
Chewie	2016-09-21	80.9	84.5	74.6	81.7	74.9	77.4	67.9	76.8	77.9	83.3	69.6	78.6
Chewie	2016-10-05	82.5	84.4	81.4	81.6	71.9	72.4	71.0	72.2	83.3	86.2	80.5	83.2
Chewie	2016-10-07	83.9	84.8	82.6	83.7	72.9	72.7	68.6	76.0	80.8	83.4	79.2	79.1
Mihili	2014-02-03	74.0	75.6	71.9	72.3	52.0	51.9	48.5	51.9	70.9	76.0	67.2	67.4
Mihili	2014-02-17	81.7	84.2	76.4	82.8	70.0	70.4	66.3	70.8	76.5	80.2	68.9	77.9
Mihili	2014-02-18	83.9	83.5	80.9	85.8	75.0	73.7	75.5	75.3	78.1	80.9	71.3	80.1
Mihili	2014-03-07	78.6	78.8	76.0	79.7	66.1	65.4	64.5	67.0	71.2	71.6	68.1	72.5

Table S 6: Variance explained by a PSID decoder given neural recordings from M1, PMd, or both areas together (R^2, %). Green values indicate cases in which PSID explained more variance than the average hand trajectory (i.e. Table. 2). Note, that PSID never explained uninstructed variance in perturbed trials.

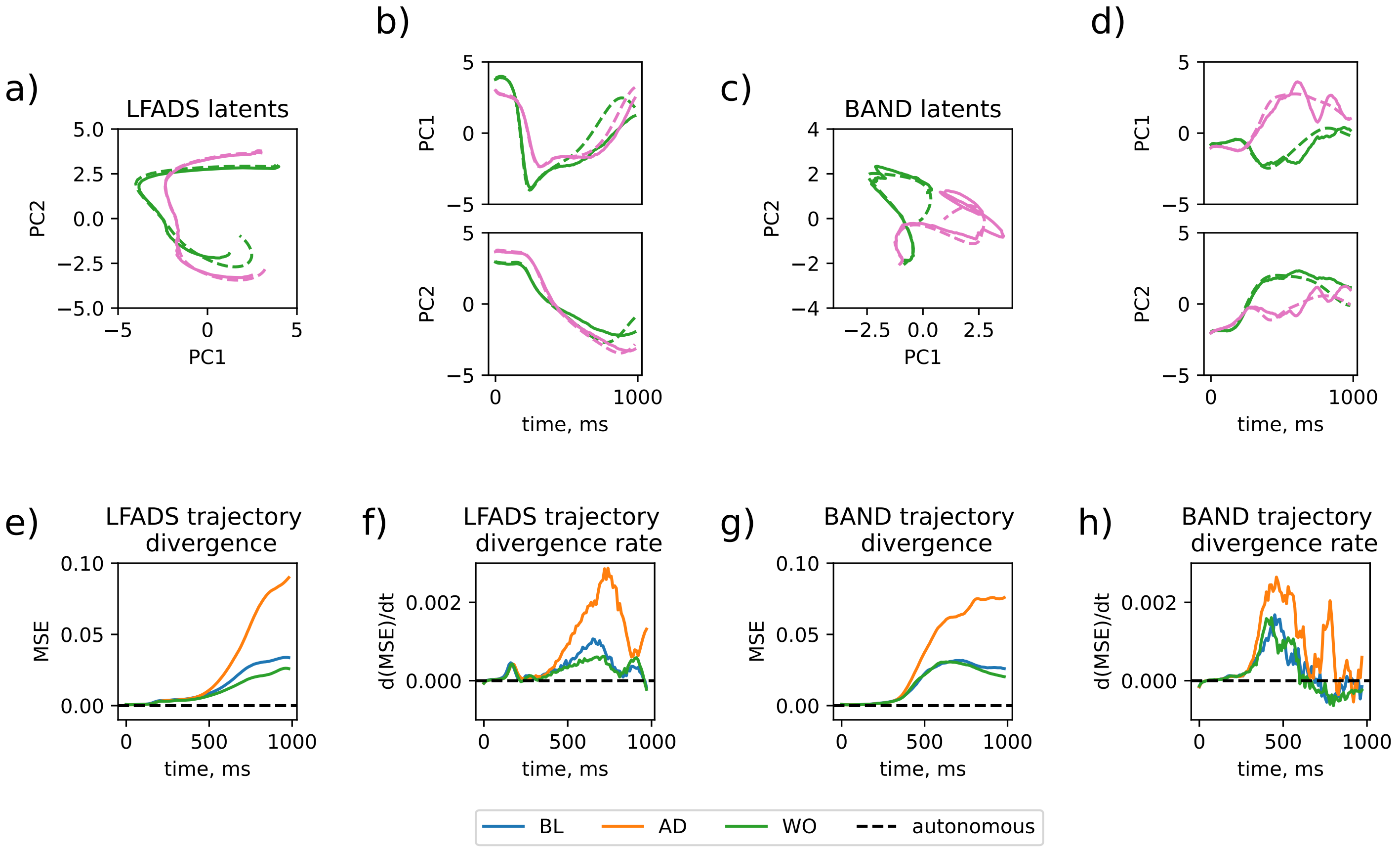

Figure S 8: Control inputs in BAND cause only a transient deviation from the autonomous dynamics trajectory. **a)** and **c)** Top two PCs of the latent space for LFADS and BAND, showing divergence between controlled (solid) and autonomous (dashed) trajectories in two example trials, corresponding to Fig. 4b; **b)** and **d)** Evolution of these two top PCs in time; **e)** and **g)** Mean-squared error (MSE) between controlled and autonomous latent trajectories in LFADS and BAND over time; **f)** and **h)** The rate of change for MSE; *Note: the data here is aligned to movement onset, as in Fig. 4-5; same trends hold true for go-cue alignment (not shown).* This figure shows that trajectories stop diverging (MSE plateaus in (g)) and even begin converging (dMSE/dt becomes negative in (h)) in BAND latent space (in all epochs), suggesting that control inputs cause only a transient deviation from an autonomous latent trajectory; this is not the case for LFADS model (see (e) and (f)), where control inputs simply refine the prediction, without adding a qualitatively different component to the neural code (specifically, correlates for hand velocity oscillations).

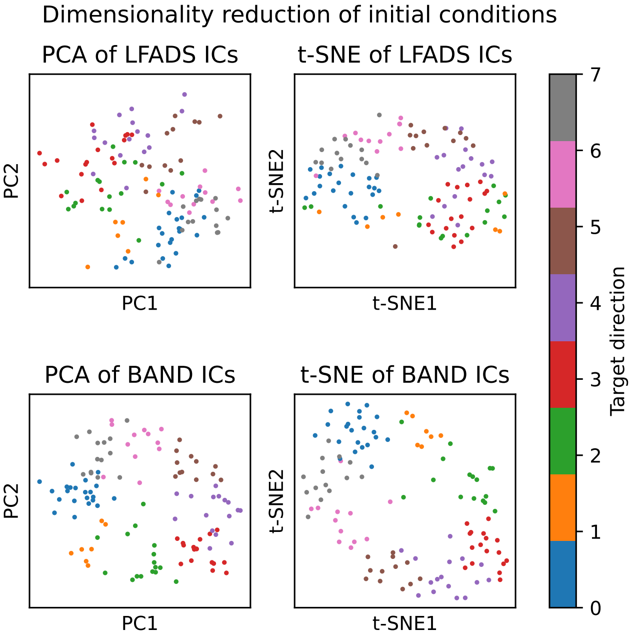

Figure S 9: Initial conditions for LFADS vs BAND (both with 8 factors). Initial conditions (ICs) in BAND are more disentangled in PC space compared to LFADS due to linear alignment of latent dynamics to the behavior. Yet, nonlinear dimensionality reduction (t-SNE) can find ring structure in both representations. Each point here – initial condition of a trial from validation set (all epochs).

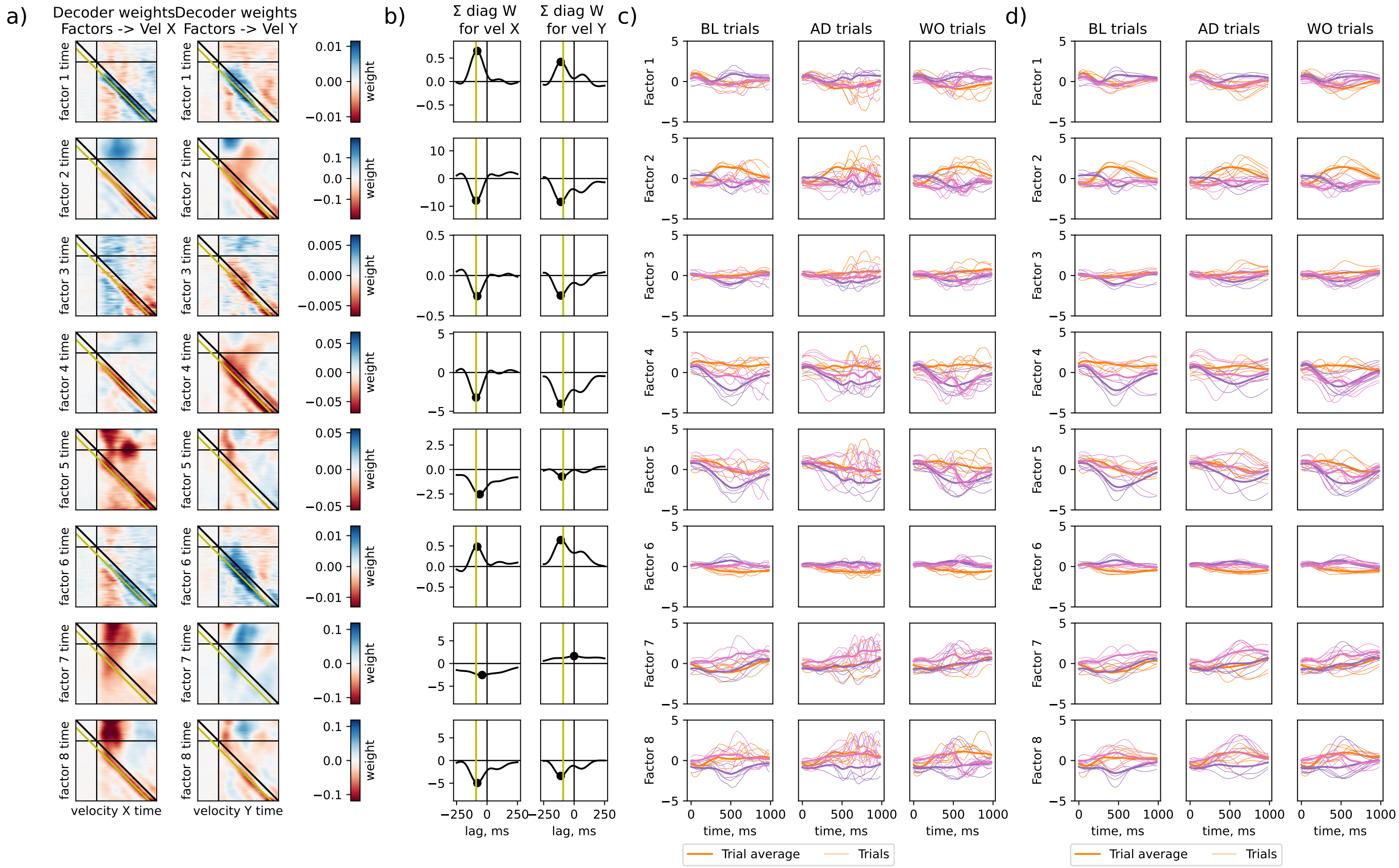

Figure S 10: Behavior decoder weights and factors for a 8-factor BAND model with a causal controller.

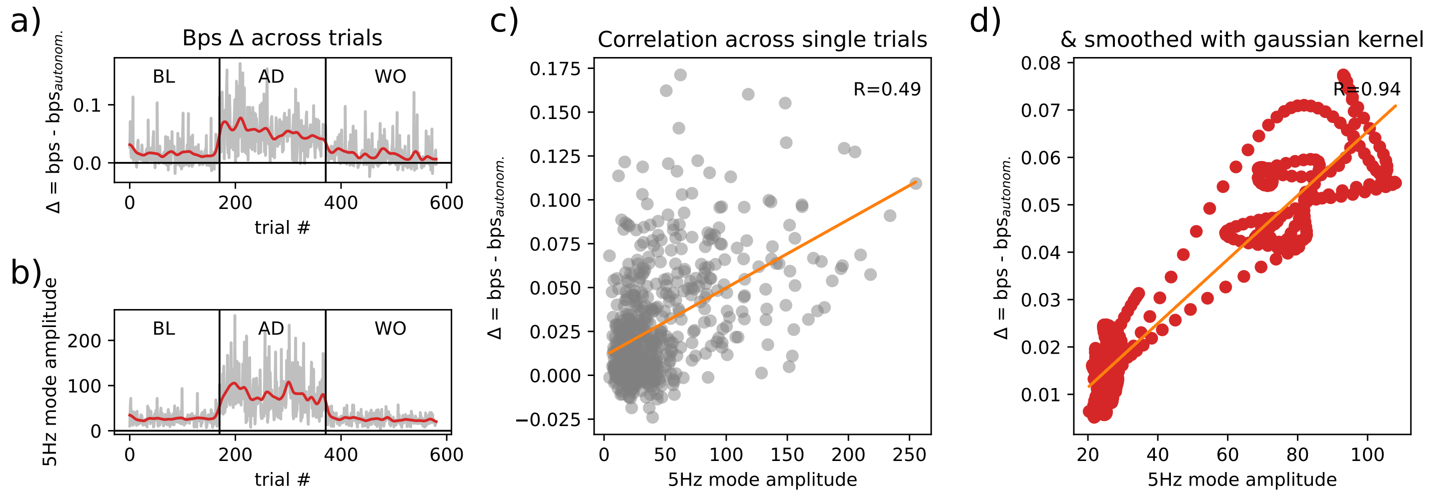

Figure S 11: The controller adds oscillations to neural factors. a) BAND controller explains more neural variability (bits / second) in adaptation trials than in other epochs without perturbation; b) amplitude of the 5 Hz hand velocity oscillations (reproduces Fig. ??e, left); c) Correlation between single trial neural variability explained by controller (bps) and amplitude of the 5 Hz hand velocity oscillation mode (R=0.49); d) Correlation between across-trials smoothed trends (gaussian kernel s.d.=5 trials) of neural variability explained by controller (bps) and amplitude of the 5 Hz hand velocity oscillation mode (R=0.94);

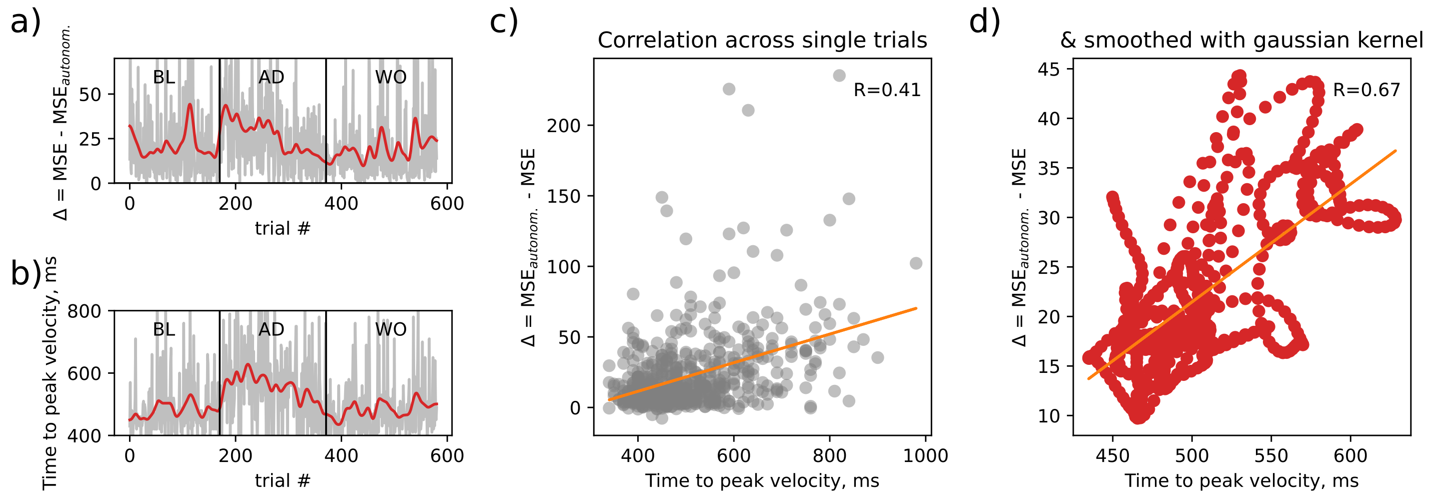

Figure S 12: The controller contributes more to behavior prediction in the trials when reaching max velocity was delayed. a) Drop in mean-squared error due to controller ablation in BAND; b) Time from the start of the trial (movement onset - 250 ms) to reaching peak velocity; c) Correlation between single trial ΔMSE and time to reach peak velocity (R=0.41); d) Correlation between across-trials smoothed trends (gaussian kernel s.d.=5 trials) of ΔMSE and time to reach peak velocity (R=0.67);
